# Shunt infection in a single institute: a retrospective study

**DOI:** 10.1186/s41016-018-0115-x

**Published:** 2018-05-07

**Authors:** Bing Qin, Gao Chen, Jingyin Chen

**Affiliations:** Department of Neurosurgery, the Second Affiliated Hospital of Zhejiang University, School of Medicine, Hangzhou, China

**Keywords:** Shunt infection, Ventriculo-peritoneal shunt, Lumbo-peritoneal shunt, Bacteria

## Abstract

**Background:**

Shunt infection (SI) is a dreaded and major complication in the management of hydrocephalus after cerebral fluid shunts. We reviewed retrospectively shunted for hydrocephalus during the last 2 years to evaluate the incidence of SI, including the risk factors and types of infection.

**Methods:**

Patients who had undergone a shunt operation from January 2013 to December 2014 in our hospital were observed, study clinical data and a 6-24 months follow-up. Patients with infection complications were found and investigated.

**Results:**

Among 343 cases of shunt surgery performed in our hospital, 6–24 months follow-up was done. 13 patients (10 men and 3 women) were found shunt infections, 11 (3.7%) were post-operation of ventriculo-peritoneal shunt and 2 (4.2%) of lumbo-peritoneal shunt.92.3% cases of shunt infections were present within 2 months after shunt surgery, gram positive cocci accounted for 90% of the bacteria. After different surgery and antibiotic treatment, 8 patients became better and 5 worse.

**Conclusions:**

The data in our single institution shows no significant differences between sex and shunt surgery. Infections more likely to present within the first 2 months after shunt placement, and gram-positive cocci account for a great proportion in detected bacteria.

## Background

Cerebral fluid shunt as one of the most common and effect treatments for hydrocephalus has last for nearly 60 years. However, various complications associated with shunt procedure have been wildly reported. Despite the advent of surgical techniques, imaging techniques, new materials and antibiotics, shunt infection is still a serious complication companion with high morbidity. Once happened, fever, meningitis, abscess, shunt dysfunction, acute hydrocephalus may present in patients, to some extent, it is another catastrophe towards patients and their family. Therapy for it is usually experimental and the outcomes differ greatly. We concluded the clinical data in a single institute in recent 2 years and reviewed previous papers.

## Methods

This retrospective study including 13 patients with infection complications secondary to shunt operations, between January 1st,2013 and December 31th,2014, in the Department of Neurosurgery from the 2nd Affiliated Hospital of Zhejiang University, School of Medicine. Bucharest 357 patients were treated with V-P shunt (ventriculo-peritoneal shunt) or L-P shunt (lumbo-peritoneal shunt), exclude 14 cases lost to follow-up, occurred in 3.8% (13/343) cases.

We collected a total of 357 patients, aged 6–84 years, with an average of 53 years, from a single center of our hospital during 2013.1.1 to 2014.12.31. There were 14 cases lost to follow-up, and the other cases were followed up for 6–24 months (13.6 months on average). There were 343 patients, aged 6–84 years old, the average age was 53.1, including 207 male and 136 female, and the average was 52.5 and 54.3 respectively. A routine follow-up of these patients was done, including 11 cases of death in 6 months after surgery and the follow-up was terminated.

We observed patients symptoms during the follow-up period, then physical examination and laboratory tests were performed on patients with abnormal symptoms due to various reasons, in order to find positive cases. Shunt infection was defined by either microbiological determination of presence of bacteria in a culture or Gram stain of CSF, wound swab, and/or pseudocyst fluid or shunt erosion (visible hardware) or abdominal pseudocyst (even without positive culture) [1], the data also include patients with apparent peritonitis or/and meningitis and obviously high white blood cell count(> 50/uL) and neutrophils proportion, decreased sugar level and increased protein level in cerebrospinal fluid. 13 cases (3.8%) were found infected at last. Our team analyzed the postoperative infection cases and studied some previous literature, tried to do some discussion.

## Results

### Groups comparison

There were 13 cases post-operation infected. The average age was 45.5 years. 10 cases are male, the rest 3 are female, the average age was 46.1 and 43.3 years old respectively. In 343 cases, 295 cases had V-P shunt, while 11 cases infected; 48 cases underwent L-P shunt, and 2 cases positive (Table 1).

**Table 1:** Comparation of different sex and different surgical method

	male			female			sum
	V-P	L-P	sum	V-P	L-P	sum	
total	180	27	207	115	21	136	343
Infected	9	1	10	2	1	3	13

### Cases data

In 13 cases of infection, there are 3 cases of pathogenic bacteria is not clear, according to the state of the cerebrospinal fluid clear infection. Among the other 10 cases, *Staphylococcus aureus* in 4 cases, epidermidis Staphylococcus in 2 cases, 1 cases of Staphylococcus capitis, 1 cases of Staphylococcus sciuri, Enterococcus faecium in 1 case, and 1 case of Baumanii. We focused on these 13 cases, the basic situation, the operation mode, the infection of bacteria, the symptoms appear, and the related treatment plan and prognosis are show in Table 2.

**Table 2:** Lists of 13 infected cases

cases	sex	age^*1^	pathography	shunt	operation before	symptom	onset time after shunt	bacteria	treatment	outcome
1	male	57	ventriclar neoplasms	V-P	resection	coma,fever	23 days	epidermidis Staphylococcus	remove shunt device	bad
2	male	65	none	V-P	none	fever	2 months	Staphylococcus sciuri	antibiotics	good
3	male	26	head injury	V-P	hematoma evacuation	fever,obstruction	5 days	*Staphylococcus aureus*	lumbar darinage	bad
4	male	39	none	V-P	external ventricular drainage	headache	22 days	not clear	take out peritoneal tube	bad
5	female	23	head injury	L-P	hematoma evacuation, lumbar drainage	fever	16 days	Baumanii	antibiotics	good
6	male	49	head injury	V-P	debridment, hematoma evacuatiom	fever	1 months	Staphylococcus capitis	remove shunt device	good
7	male	45	head injury	V-P	none	peritonitis,headache	4 months^*2^	not clear	remove shunt device	good
8	female	51	none	V-P	none	shunt exposed,pus	2 months	not clear	debridment	good
9	male	34	head injury	V-P	hematoma evacuatiom	fever,obstruction	6 days	Staphylococcus anreus	lumbar darinage	bad
10	male	64	head injury	V-P	hematoma evacuatiom	fever,peritonitis	8 days	Enterococcus faecium	remove shunt device,ventriculo-artial shunt	good
11	male	43	cerebellar hemorrhage,AVM	V-P	external ventricular drainage,posterior fossa decompression	fever	10 days	Staphylococcus aureus	antibiotics	bad
12	male	37	head injury	L-P	hematoma evacuatiom	headache	2 months	Staphylococcus aureus	remove shunt device,debridment,V-P	good
13	female	54	none	V-P	none	fever,shunt exposed	20 days	epidermidis Staphylococcus	remove shunt device	good

*1: The unit for age is year

*2: In this case, an appendicitis was diagnosed and underwent an appendectomy 10 days before the onset time

## Discussion

Although much development has been made in neuroendoscopic surgery, the most common treatment for hydrocephalus remains shunt. We collected data in our unit to show the clinical characteristics of infection complications secondary to shunt operations. In 357 cases collected, 350 are over 16 years old, additionally, all the 13 infected cases are adults.

Bacteria can track along the catheter into the brain, hence, the brain is susceptible to infection after shunt placement. Recently, some researches showed antibiotic-impregnated shunt catheters can reduce the infection rate significantly, but still controversy. Postoperative shunt infections were reported occur in 0.3–17% [2, 3] of cases in most neurosurgical institutes.

In our unit, V-P shunt and L-P shunt are routine surgical options, we noticed there is no significant differences in age, gender, and infective complication between the groups underwent different operations (Table 1). V-P shunt is the most common treatment for hydrocephalus, but a large number of reports suggest that L-P shunt is an effective shunting procedure in communicating hydrocephalus. But there is no study compelling enough to prove which shunt surgery has less complications, so if L-P shunt can be an alternative to V-P shunt remains controversial [4, 5].

We noticed that post-operation of head trauma seems more likely to be infected. In our study, head trauma before shunt makes a rate of 53.8% (7/13) of infected cases. They all had underwent an operation for injury, sometimes debridement operation was needed when there was an open wound. In 2 cases, other shunt surgery was done before the shunt operation in our hospital. So undergoing surgery more recently may cause more easily to suffer an infection in the following shunt, we speculate the CSF not clear enough is the main reason.

Most cases of shunt infections are present within the first 2 months (up to 92.3%) after the shunt surgery. This situation is similar to other reports by different authors. Atiqur Rehman introduces their study in which 10 cases appear infection in 111 post operation of V-P shunt cases, the clinical symptoms appears in 2 months accounts 70% [6]. Florian and Fried aim that infections symptomatic rapidly after shunt insertion, 70% of them being diagnosed within the first month. By 9 months 90% of shunt infection became clinical manifested [7, 8]. We suggest that define 2 months after the shunt operation as an early stage to make a more close follow up. The infection cases in this stage are usually a surgical related infection, often appears a fever, headache, obstruction, and need to remove the shunt devices and use antibiotics.

There is no persuasive guideline to shed light on the timing and whether to remove the shunt device once the infection happened [9]. Scheffler et al. [10] compared three approaches in cure rate, morbidity and mortality, suggest that shunt removal, external ventricular drainage placement or ventricular taps and antibiotics, followed by creation of a new shunt when CSF sterility is achieved, is the most effective method of treatment for CSF shunt infection. In our study, 83.3% (5/6) has a good outcome after totally removing catheters, in contrast, only 42.9% (3/7) has a good outcome while not completely removed shunt devices.

Gram positive cocci were the most common bacteria in infective complication once a shunt is placed. In 10 cases which pathogenic bacteria are clearly identified, gram positive cocci accounted for 90% (9/10), and 80% (8/10) is staphylococcus. These bacteria are parasitic on the skin, which is very easy to be brought into the CSF or adhesive in the shunt device, sometimes in the catheters or the valves. As a result, the infection symptoms appear in a very short time after the shunt surgery in these cases.

The treatment of infective complication typically includes systemic intravenous antimicrobials, 13 cases were all given intravenous antimicrobials. Gram positive cocci has a relatively high morbidity of infection, so we experimentally started out with vancomycin (0.5–1.0 g twice a day), or linezolid (0.6 g twice a day) in instead. Sometimes cephalosporins carbapenems was used as a combination drug. Some authors suggest using intraventricular antibiotics as while intraventricular vancomycin has a superior safety profile with no clinically significant toxicity reported [11]. A study show significantly reduces infection rates after intraventricular antibiotics treatment compared with systemic use alone, they reported a 93% success rate in treating CSF shunt-associated infections caused by coagulasenegative Staphylococcus with intraventricular vancomycin and systemic therapy alone, without surgical intervention [12].

In our hospital, we have a general operating procedures may help reduce infection: preoperative antibiotics, double gloving and the shortest exposure time of shunt devices----which means the operation presents skull drilling to dural or open to lumbar spinous process, abdomen incised to the peritomeum and establish the subcutaneous sinus for shunt tube, at last take out the shunt devices to perform the ventricular or lumbar puncture and place the tube into the abdomen. Recently, many studies spare no effort in how to avoid a shunt-associated infection or reduce the infect rate. They suggest methods to reduce infection including preoperative systemic antibiotics and specific surgical protocol. This includes limited access to the operating room and performing the procedure in the early morning to reduce bacterial presence in the environment. To minimize contamination, a so-called no-touch technique is adopted with antibiotic solution wound irrigation, double gloving [6], and antibiotic-impregnated shunt usage [13]. However, whether the antibiotic-impregnated shunt is effective still has a lot of controversy. There are evidences as well to discredit the benefits of antibiotic-impregnated shunt devices [3, 14]. Further investigation is still needed.

## Conclusions

The overall date, which contains 357 cases and a two-year follow-up, shows no significant differences between sex and shunt surgery. In this single institution review, we find that SI is more likely to present within 2 months after procedure is made, so we propose that this period as an early stage to have an intensified observation. Gram-positive cocci is the most common bacteria found in infected patients after the placement of a shunt, our results showed gram positive cocci accounted for 90% (9/10), and 80% (8/10) is staphylococcus.

## Abbreviations

CSF: Cerebrospinal fluid
L-P shunt: Lumbo-peritoneal shunt
V-P shunt: Ventriculo-peritoneal shunt
